# Association between serum liver enzymes and hypertension using propensity score matching analysis: evidence from a large kurdish prospective cohort study

**DOI:** 10.1186/s12872-022-02884-3

**Published:** 2022-11-10

**Authors:** Mina Tahmasebi Fard, Farid Najafi, Shahab Rezaeian, Maryam Kohsari, Mehdi Moradinazar

**Affiliations:** 1 Department of Epidemiology, School of Health, Kermanshah University of Medical Sciences, Kermanshah, Iran; 2 Behavioral Diseases Research Center, Kermanshah University of Medical Sciences, Kermanshah, Iran; 3 Student Research Committee, Kermanshah University of Medical Sciences, Kermanshah, Iran; 4 Infectious Diseases Research Center, Kermanshah University of Medical Sciences, Kermanshah, Iran

**Keywords:** Hypertension, Propensity score matching, Liver enzymes (GGT, ALT, AST, ALP)

## Abstract

**Background:**

The association between liver enzymes and hypertension (HTN) has been reported in some studies and the findings are inconsistent. This study was conducted to evaluate the association of liver enzymes with HTN among the Iranian Kurdish population.

**Methods:**

This prospective cohort study was a part of the 5-years (2017–2021) follow-up phase of the Ravansar Non-Communicable Disease (RaNCD) cohort study in Kermanshah province, western Iran.The association between alanine aminotransferase (ALT), aspartate aminotransferase (AST), gamma-glut amyl transferase (GGT), and alkaline phosphatase (ALP) and HTN was investigated by Cox proportional-hazard model (CPHM). We used one-to-one Propensity score matching (PSM) analysis to minimize the effects of confounding factors on the relationship between liver enzymes and HTN .

**Results:**

The full population included a total of 8267 participants. According to PSM, for liver enzyme GGT a total of 3664 participants were analyzed. The results of multivariate CPHM showed there is a relationship between participants with high level of GGT and had a higher risk of HTN (HR 1.34; 95% CI: 1.11–1.63). After PSM analysis, the effect of GGT on HTN remained positive and significant (HR 1.48; 95% CI: 1.22–1.78). The 5-years incidence rate of HTN in men and women were 1.27 and 0.81 (person-year), respectively.GGT had the greatest accuracy, which demonstrated an AUROC of 0.7837.

**Conclusion:**

Results of this study showed GGT could be a potential biomarker among liver enzymes for early detection of HTN. Therefore, monitoring GGT levels is helpful in the early detection of HTN.

**Supplementary Information:**

The online version contains supplementary material available at 10.1186/s12872-022-02884-3.

## Background

Hypertension (HTN) is one of the most important risk factors for cardiovascular disease (CVDs) across the world. Usually, HTN has no symptoms, and various causes developing it, but it can be controlled with medications [1]. The prevalence of HTN in different countries varies from 10% to more than 60% [2], and its average global prevalence is reported about 22% in 2021 [3]. Based on the evidence, the prevalence of HTN will be increased to 29.2% in 2025 [4].

Risk factors for HTN are divided into unmodifiable factors, such as gender, age, and family history, and modifiable factors, such as obesity, sedentary lifestyle, stress, poor diet, and etc. [2, 5–7]. According to previous studies, there is a significant association between age, smoking, obesity, high-calorie diet, salt intake, sedentary lifestyle, literacy, and alcohol with HTN [8–12]. However, the role of liver enzymes as a risk factor for HTN has not been well known [13]. The serum levels of the liver enzymes alanine aminotransferase (ALT), gamma-glutamyl transferase (GGT), aspartate aminotransferase (AST), and alkaline phosphatase (ALP) are markers of liver function [14], and also elevated in liver dysfunctions and other metabolic disorders [15, 16]. GGT is used to assess alcohol abuse and oxidative stress [17]. Aminotransferases (ALT and AST) play an important role in liver gluconeogenesis and amino acid metabolism [18, 19]. ALP utilize for detection gallbladder and bile duct disorders [20].

There are limited studies on the relationship between liver function and HTN incident. Some study such as Taiwan cohort study and study on Bangladeshi population, found that elevated GGT and ALT levels were associated with the HTN [13, 21]. In Iranian population evidence illustrated that HTN has become one of the leading causes of mortality and Disability-adjusted life years (DALYs), and prevalence rates of HTN are increasing (overall rate 25% for HTN) [22]. Hence, determining new factors that help timely diagnose can considerably reduce the mortality and DALYs rate associated with HTN.

In the previous study, through a cross-sectional survey on the initial phase of the RaNCD cohort, we established that elevated serum levels of GGT and ALP could increase the incidence risk of HTN[23]. To confirm our prior results, we conduct a five-year follow-up study on the same population in the present study. In this study, we used PSM analysis to overcome the effect of possible selection bias and to further control for potential confounding factors. PSM is a tool to adjust a treatment effect for measured confounders and is so an alternative to common regression adjustment. PSM first was used by Rosenbaum and Rubin in 1983, to reduce bias in an observational study. PSM analysis tries to compare outcomes between patients who have similar distributions of all covariates measured, therefore clarify variable’s effects on outcome[24].

## Methods

### Study design and participants

This prospective cohort study was a part of the 5-years (2017–2021) follow-up phase of the Ravansar Non-Communicable Disease (RaNCD) cohort study in Kermanshah University of Medical Sciences (KUMS), western Iran. The RaNCD cohort study, as a part of the PERSIAN (Prospective Epidemiological Research Studies in Iran) Cohort, focus on Ravansar constant inhabitants aged 35–65 years of old. All 21 PERSIAN cohort studies used the same questionnaire and aim to follow up participants for at least 15 years after enrollment. Ravansar, in western Iran, is a city in Kermanshah province close to Iraqi borders. Its residents are mostly Kurdish. The design and foundations of the PERSIAN cohort study were detailed here [25].

### Inclusion and exclusion criteria

Inclusion criteria of RaNCD cohort study were residency more than one year in that city, people aged 35–65 years who live at least 9 months of the year in the area, persons who were willing to participate and complete the study, people formally consent to participate and were able to communicate with the research team. In this study, people had liver disease and/ or used liver medications (n = 210), who had high blood pressure and/ or used antihypertensive drugs (n = 1558), participants in special health status such as cancer or pregnancy (n = 30) and those who were unwilling to participate in the follow-up were excluded to eliminate confounding variables. Finally, out of 10,065 participants in the cohort study, 8267 persons were enrolled in the present study.

### Data collection and quality control

Data were collected by our research team at the Cohort Center, who were well trained in study protocols for patient entry and data gathering. Demographic data, lifestyle risk factors, medical history, and medication use for previous or current underlying diseases were obtained with standardized questionnaires. All completed questionnaires were checked and verified for errors by the quality control team before final analysis. The patients’ national identification numbers were used to avoid duplicate follow up.

### Definition and measurements

All participants were advised to fast for about 10–12 h. About 5 ml of blood was drawn intravenously and obtained with a venoject tube. The serum was centrifuged at 300 g for 10 min and stored at -20 °C until tested. Liver enzymes (including ALT, AST, GGT, and ALP) were analyzed with an enzymatic colorimetric assay by Mindray-BS-380 auto analyzer (Mindray, USA). In this study, Youden’s index was used to determine the best cut point of liver enzymes, this points had highest sensitivity and specificity for each liver enzymes. Best cut points were AST > 20.0 U/L, ALT > 21.3 U/L, GGT > 21.8U/L, and ALP > 194 U/L.

Bio impedance Analyzer (BIA) (In Body 770 Bio space, Korea) was used to assess the anthropometric measurements. Height was measured with 0.1 cm accuracy with a stadiometer and weight was measured with 0.5 kg accuracy. Body mass index (BMI) was computed as body weight (kg) divided by height (m2). Waist circumference (WC) was assessed according to cm around the middle of the body at the upper part of the hip bones.

HTN was measured through a standardized procedure after 5 min of rest with two measurements of the right arm and two measurements of the left arm with cuff size adjusted to arm circumference. The cuff was placed on the arm at the heart level using a Riester duplex blood pressure device. There was at least a one-minute interval between two separate measurements. The average of two measurements for each arm was calculated. The higher measurement of two arms was considered the mean of systolic blood pressure (SBP) and diastolic blood pressure (DBP) [26]. participants with SBP ≥ 140 mm Hg and/or DBP ≤ 90 mm Hg and/or taking medicine according to the doctor’s prescription as hypertensive persons.

### Follow up

**Fig. 1:**
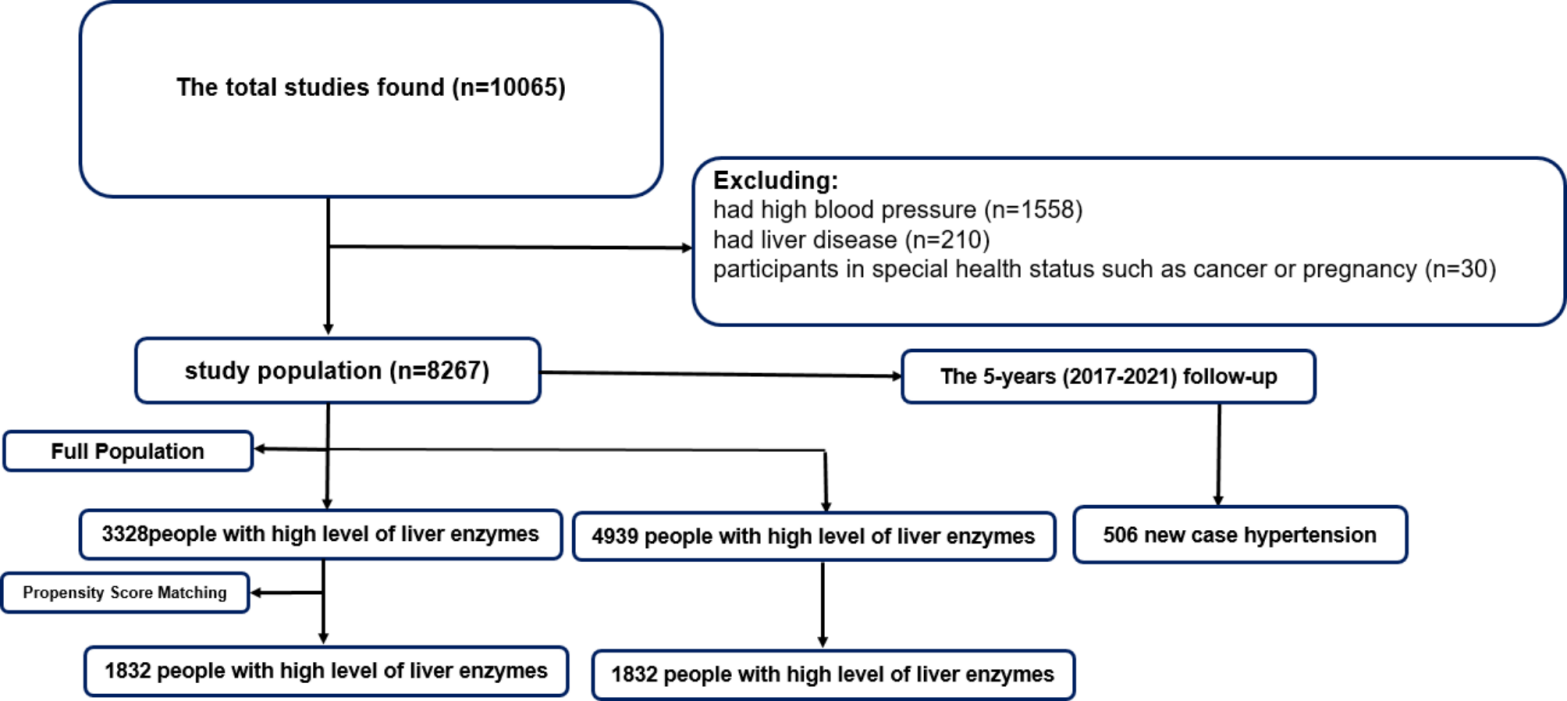
Flowchart of study design

During follow-up phase, telephone-based questionnaires including the occurrence of death or the incidence of any medical events, hospitalizations, or diagnostic/therapeutic care will be annually completed for all participants. Also, disease registration centers reports will be collected if a participant or his/her family does not answer six phone calls in two weeks, research team will follow the phone call with a house visit to perform a face-to-face interview. If a participant has been detected with a main NCD, research team obtains copies of medical documents for further assessment and recording. If needed, medical/physical examinations are applied to determine a diagnosis. Likewise, medical events forms will be completed. In the event of death, a verbal autopsy specialized from for the Iranian population is also completed Fig. 1.

### Statistical analysis

Based on the review of the literature in this field, 21 possible variables that may affect the relationship between liver enzymes and the incidence of hypertension were identified, these variables are a group of confounding variables that include: age; gender; education years; physical activity; alcohol consumption; body mass index; smoking status; diabetes melitus; cardiovascular disease; salt use; family medical history; cholesterol; triglyceride; high-density lipoprotein; low-density lipoprotein; residence type; socio-economic status; healthy nutrition index; depression; job; used oil type. Which was used for adjusted in the full population and PSM, for more information on how to segment and measure these variables, you can refer to RaNCD Cohort protocol [25, 27]. Incidence rate was calculated by dividing the number of new cases of HTN by the population at risk (person - year).

In this study, PSM was performed using a 1:1 matching protocol without replacement (greedy-matching algorithm/nearest neighbor matching), thus participants with high levels of liver enzymes as the exposure group were matched with participants with low levels of liver enzyme as the non-exposure group. We also investigated the relationship between liver enzymes and the hypertension in the full population using a Cox proportional-hazard model (CPHM), and P-value < 0.05 were considered significant.

The Akaike information criterion (AIC) and Bayesian information criterion (BIC) were used to compare the goodness of model fit for hypertension prediction. The Nagelkerke pseudo-R2 and the area under the receiver-operating characteristic curve (AUROC) were calculated to compare the regular differences in the distribution of variables between two groups of the study. Decreasing the level of all mentioned indices is indicated the goodness of choosing a model with low regular differences. We analyzed all data using STATA software version 14.2.

#### Ethical approval

The Research Ethics Committee at KUMS approved the study protocol (Ethics No. IR.KUMS.REC.1399.1168). Also, patients were informed about participating in the study and signed the consent form. Patient data were kept confidential with the access limited to two of researchers and the quality control physician.

## Results

The full population included a total of 8267 participants (before excluding participants with missing covariate data): 4939 in the unexposed group (people with low level of liver enzymes) and 3328 in the exposed group (people with high level of liver enzymes). According to PSM, a total of 1832 unexposed people were matched to 1832 exposed people. The baseline characteristics (demographic and biochemical markers) and adjusted variables of the matched and unmatched sample are displayed in Table 1, and in Supplementary Table 1 **(S1)** Basic demographic data of the study population, based on liver enzymes and variables also Basic biochemical variables of the study population are showen in Table 2. After 5-years follow-up, 506 participants developed hypertension. The 5-years incidence rate of HTN in men and women were 1.27 and 0.81 (person-year), respectively.

**Table 1:** Basic demographic data of the study population [n (%)] base on liver enzymes

Variables	GGT				ALT					AST						ALP			
	**Full population**		**Propensity score matched**		**Full population**		**Propensity score matched**			**Full population**			**Propensity score matched**			**Full population**		**Propensity score matched**	
	Un-*** expose	Expose**	**Un expose**	**Expose**	**Un-** **expose**	**Expose**	**Un-** **expose**	**Expose**		**Un-** **expose**		**Expose**	**Un-** **expose**		**Expose**	**Un- expose**	**expose**	**Un- expose**	**expose**
	4939	3328	1832	1832	4266	4001	1852	1852		4458		3809	2047		2047	4555	3712	2168	2168
Age* group n (%)																			
35–45	2676 (54.2)	1671 (50.2)	887 (48.4)	920 (50.2)	2149 (50.4)	2198 (54.9)	936 (50.5)	1017 (54.9)		2364 (53.0)		1983 (52.1)	994 (48.6)		1065 (52.0)	2601 (57.1)	1746 (47.0)	1004 (46.3)	1020 (47.0)
46–55	1481 (30.0)	1085 (32.6)	606 (33.1)	597 (32.6)	1339 (31.4)	1227 (30.7)	611 (33.0)	568 (30.7)		1373 (30.8)		1193 (31.3)	675 (32.9)		642 (31.4)	1360 (29.9)	1206 (32.5)	755 (34.8)	704 (32.5)
56–56	782 (15.8)	572 (17.2)	339 (18.5)	315 (17.2)	778 (18.2)	576 (14.4)	305 (16.5)	267 (14.4)		721 (16.2)		633 (16.6)	378 (18.5)		340 (16.6)	594 (13.0)	760 (20.5)	409 (18.9)	444 (20.5)
Gender *																			
Mele	1857 (37.6)	2102 (63.2)	1124 (61.4)	1157 (63.2)	1351 (31.7)	2608 (65.2)	1167 (63.0)	1207 (65.2)		1589 (35.6)		2370 (62.2)	1267 (61.9)		1273 (62.2)	2058 (45.2)	1901 (51.2)	1142 (52.7)	1110(51.2)
Female	3082 (62.4)	1226 (36.8)	708 (38.6)	675 (36.8)	2915 (68.3)	1393 (34.8)	685 (37.0)	645 (34.8)		2869 (64.4)		1439 (37.8)	780 (38.1)		774 (37.8)	2497 (54.8)	1811 (48.8)	1026 (47.3)	1058(48.8)
Education Years																			
0	1164 (23.6)	633 (19.0)	370 (20.2)	348 (19.0)	1141 (26.8)	656 (16.4)	335 (18.1)	304 (16.4)		1003 (22.5)		794 (20.8)	400 (19.5)		427 (20.9)	863 (18.9)	934 (25.2)	487 (22.5)	545 (25.1)
1–5	2028 (41.1)	1177 (35.4)	656 (35.8)	648 (35.4)	1811 (42.4)	1394 (34.8)	662 (35.7)	645 (34.8)		1875 (42.1)		1330 (34.9)	759 (37.1)		715 (34.9)	1853 (40.7)	1352 (36.4)	862 (39.8)	790(36.5)
6–9	835 (16.9)	624 (18.7)	347 (19.0)	344 (18.8)	677 (15.9)	782 (19.5)	358 (19.3)	362 (19.6)		763 (17.1)		696 (18.3)	412 (20.1)		373 (18.2)	798 (17.5)	661 (17.8)	360 (16.6)	386 (17.8)
10–12	573 (11.6)	556 (16.7)	266 (14.5)	306 (16.7)	419 (9.8)	710 (17.8)	292 (15.8)	329 (17.8)		524 (11.7)		605 (15.9)	298 (14.6)		326 (15.9)	647 (14.2)	482 (13.0)	276 (12.7)	282 (13.0)
13<	339 (6.8)	338 (10.2)	193 (10.5)	186 (10.1)	218 (5.1)	459 (11.5)	205 (11.1)	212 (11.4)		293 (6.6)		384 (10.1)	178 (8.7)		206 (10.1)	394 (8.7)	283 (7.6)	183 (8.4)	165 (7.6)
Physical Activity METs																			
Low	1301 (26.3)	1127 (33.9)	636 (34.7)	621 (33.9)	1123 (26.3)	1305 (32.6)	642 (34.7)	604 (32.6)		1306 (29.3)		1122 (29.3)	621 (30.0)		603 (29.4)	1288 (28.3)	1140 (30.7)	674 (31.1)	666 (30.7)
Medium	2509 (50.8)	1448 (43.5)	775 (42.3)	797 (43.5)	2237 (52.5)	1720 (43.0)	764 (41.2)	797 (43.0)		2287 (51.3)		1670 (43.8)	863 (42.2)		898 (43.9)	2227 (48.9)	1730 (46.6)	1003 (46.3)	1010(46.6)
Intense	1129 (22.9)	753 (22.6)	420 (23.0)	414 22.6)	906 (21.2)	976 (24.4)	446 (24.1)	451 (24.1)		865 (19.4)		1017 (26.7)	563 (27.5)		546 (26.7)	1040 (22.8)	842 (22.7)	491 (22.6)	492 (22.7)
Alcohol Consumption																			
No	4761 (96.4)	3092 (92.2)	1711 (93.4)	1702 (92.9)	4117 (96.5)	3736 (93.4)	1736 (93.7)	1729 (93.4)		4251 (95.4)		3602 (94.6)	1933 (94.4)		1936 (94.6)	4355 (95.6)	3498 (94.2)	2042 (94.2)	2043 (94.2)
Yes	178 (3.6)	236 (7.1)	121 (6.6)	132 (7.1)	149 (3.5)	265 (6.6)	116 (6.3)	123 (6.6)		207 (4.6)		207 (5.4)	114 (5.6)		111 (5.4)	200 (4.4)	214 (5.8)	126 (5.8)	125(5.8)
BMI*																			
18.9>	166 (3.4)	40 (1.2)	22 (1.2)	24 (1.3)	169 (4.0)	37 (0.9)	34 (1.8)	17 (0.9)		113 (2.5)		93 (2.4)	57 (2.8)		50 (2.4)	128 (2.8)	78 (2.1)	41 (1.9)	46 (2.1)
19-24.9	1654 (33.5)	682 (20.5)	375 (20.5)	434 (23.7)	1427 (33.5)	909 (22.7)	464 (40.925.1)	421 (22.7)		1228 (27.6)		1108 (29.1)	633 (30.9)		596 (29.1)	1383 (30.4)	953 (25.7)	559 (25.8)	557(25.7)
25-29.9	2014 (40.8)	1581 (47.5)	870 (47.5)	825 (45.0)	1636 (38.3)	1959 (49.0)	817 (44.1)	906 (49.9)		1874 (42.0)		1721 (45.2)	888 (43.4)		924 (45.1)	1927 (42.3)	1668 (44.9)	954 (44.0)	974(44.9)
30.0-34.9	866 (17.5)	818 (24.6)	450 (24.6)	382 (20.8)	799 (18.7)	885 (22.1)	396 (21.4)	98 (5.3)		952 (21.4)		732 (19.2)	364 (17.8)		394 (19.3)	889 (19.5)	795 (21.4)	475 (21.9)	464 (21.4)
35.00<	239 (4.8)	207 (6.2)	114 (6.2)	168 (9.2)	235 (5.5)	211 (5.3)	141 (7.6)	20 (1.0)		291 (6.5)		155 (4.1)	105 (5.1)		83 (4.1)	228 (5.0)	218 (5.9)	139 (6.4)	127 (5.9)
Smoking status																			
Non-smoker	2142 (43.4)	1257 (37.8)	684 (37.3)	692 (37.8)	1806 (42.3)	1593 (39.8)	657 (35.5)	738 (39.9)		1875 (42.1)		1524 (40.0)	735 (35.9)		819 (40.1)	1977 (43.4)	1422 (38.3)	865 (40.0)	83 (38.3)
recently	515 (10.4)	486 (14.6)	288 (15.7)	268 (14.6)	503 (11.8)	498 (12.5)	363 (19.6)	230 (12.4)		535 (12.0)		466 (12.2)	380 (18.6)		250 (12.2)	451 (9.9)	550 (14.8)	252 (11.6)	321 (14.8)
history	316 (6.4)	331 (9.9)	175 (9.6)	182 (9.9)	263 (6.2)	384 (9.6)	167 (9.0)	178 (9.6)		300 (6.7)		347 (9.1)	200 (9.8)		187 (9.1)	342 (7.5)	305 (8.2)	210 (9.7)	178 (8.2)
the past	1966 (39.8)	1254 (37.7)	685 (37.4)	690 (37.7)	1694 (39.7)	526 (38.1)	665 (35.9)	706 (38.1)		1748 (39.2)		1472 (38.7)	732 (35.7)		791 (38.6)	1785 (39.2)	1435 (38.7)	840 (38.7)	838 (38.7)
DM incidence																			
No	4864 (98.5)	3172 (95.3)	1750 (95.5)	1746 (95.3)	4165 (97.6)	3871 (96.8)	1769 (95.5)	1792 (96.8)		4324 (97.0)		3712 (97.4)		1993 (97.4)	1995 (97.5)	4459 (97.9)	3577 (96.4)	2097 (96.7)	2089(96.4)
yes	75 (1.5)	156 (4.7)	82 (4.5)	86 (4.7)	101 (2.4)	130 (3.2)	83 (4.5)	60 (3.2)		134 (3.0)		97 (2.6)		54 (2.6)	52 (2.5)	96 (2.1)	135 (3.6)	71 (3.3)	79 (3.6)
CVD incidence																			
No	136 (2.7)	98 (3.1)	61 (2.9)	65 (3.1)	4132 (96.9)	3902 (97.5)	1802 (97.3)		1806 (97.5)		4324 (97.0)	3710 (97.4)		1993 (97.4)	1994 (97.4)	4454 (97.8)	3580 (96.4)	2097 (96.7)	2091 (96.4)
yes	4936 (97.3)	3097 (96.9)	2080 (97.1)	2076 (96.9)	134 (3.1)	99 (2.5)	50 (2.7)		46 (2.5)		134 (3.0)	99 (2.6)		54 (2.6)	53 (2.6)	101 (2.2)	132 (3.6)	71 (3.3)	77 (3.6)
Salt Use																			
Yes	1397 (28.3)	937 (28.1)	488 (26.7)	516 (28.2)	1214 (28.5)	1119 (28.0)	508 (27.4)		518 (28.0)		1269 (28.5)	1065 (28.0)		561 (27.4)	572 (27.9)	1312 (28.8)	1022 (27.5)	592 (27.3)	597 (27.5)
Sometimes	1590 (32.2)	1030 (31.0)	609 (33.2)	567 (30.9)	1352 (31.7)	1267 (31.7)	603 (32.6)		586 (31.6)		1441 (32.3)	1179 (30.9)		660 (32.2)	634 (31.0)	1448 (31.8)	1172(31.6)	685 (31.6)	684 (31.6)
No	1952 (39.5)	1361 (40.9)	735 (40.1)	749 (40.9)	1696 (39.8)	1615 (39.8)	741 (40.0)		748 (40.4)		1748 (39.2)	1565 (41.1)		826 (40.4)	841 (41.1)	1795 (39.4)	1518 (40.9)	891 (41.1)	887 (40.9)
FH1_Hypertension																			
NO	2557 (51.8)	1661 (49.9)	912 (49.8)	914 (49.9)	2183 (51.2)	2035 (50.9)	930 (50.2)		942 (50.9)		2263 (50.8)	1995 (51.3)	1034 (50.5)		1051 (51.3)	2320 (50.9)	1898 (51.1)	1110 (51.2)	1108 (51.1)
yes	2382 (48.2)	1667 (50.1)	920 (50.2)	918 (50.1)	2083 (48.8)	1966 (49.1)	922 (49.8)		910 (49.1)		2195 (49.2)	1854 (48.7)	1013 (49.5)		996 (48.7)	2235 (49.1)	1814 (48.9)	1058 (48.8)	1059 (48.9)

GGT: gamma-glutamyl transferase; ALT: alanine aminotransferase; AST: aspartate aminotransferase; ALP: alkaline phosphatase; BMI: body mass index; DM incidence: diabetes mellitus incidence; CVD incidence: cardiovascular disease incidence; FH1_Hypertension: Family medical history

*P-value < 0.005 **Expose: People with high liver enzymes ***Unexposed: People with Low liver enzymes

**Table 2:** Basic biochemical variables of the study population base on liver enzymes

Variables	GGT				ALT				AST				ALP			
	**Ful** **l population**		**Propensity score matched**		**Full population**		**propensity score matched**		**Full population**		**propensity score matched**		**Full population**		**propensity score matched**	
	Un*** expose	Expose**	**Un expose**	**Expose**	**Unexpose**	**Expose**	**Unexpose**	**Expose**	**Unexpose**	**Expose**	**Unexpose**	**Expose**	**Un expose**	**expose**	**Un expose**	**expose**
	4939	3328	1832	1832	4266	4001	1852	1852	4458	3809	2047	2047	4555	3712	2168	2168
CHOL																
Optimal	3720 (75.3)	2032 (61.1)	1146 (62.5)	1119 (61.1)	3081 (72.3)	2671 (66.8)	1193 (64.4)	1237 (66.8)	3211 (72.0)	2541 (66.7)	1379 (67.4)	1367 (66.8)	3395 (74.5)	2357 (63.5)	1402 (64.7)	1377 (63.5)
Border	920 (18.6)	943 (28.3)	463 (25.3)	519 (28.3)	876 (20.5)	987 (24.7)	460 (24.8)	457 (24.7)	945 (21.2)	918 (24.1)	482 (23.5)	493 (24.1)	889 (19.5)	974 (26.2)	553 (25.5)	569 (26.3)
High risk	299 (6.1)	353 (10.6)	223 (12.2)	194 (10.6)	309 (7.2)	343 (8.5)	199 (10.8)	158 (8.5)	302 (6.8)	350 (9.2)	186 (9.1)	187 (9.1)	271 (6.0)	381 (10.3)	213 (9.8)	222 (10.2)
TG																
Optimal	3891 (78.8)	1851 (55.6)	1046 (57.1)	1019 (55.6)	3324 (77.9)	2418 (60.4)	1126 (60.8)	1119 (60.4)	3290 (73.8)	2452 (64.4)	1367 (66.8)	1319 (64.4)	3404 (74.7)	2338 (63.0)	1415 (65.3)	1365 (63.0)
Border	622 (12.6)	668 (20.1)	368 (20.1)	368 (20.1)	524 (12.3)	766 (19.2)	316 (17.0)	355 (19.2)	634 (14.2)	656 (17.2)	334 (16.3)	353 (17.3)	603 (13.3)	687 (18.5)	370 (17.1)	401 (18.5)
Top	411 (8.3)	773 (23.2)	383 (20.9)	426 (23.2)	400 (9.4)	784 (19.6)	366 (19.8)	363 (19.6)	510 (11.5)	674 (17.7)	325 (15.9)	362 (17.7)	524 (11.5)	660 (17.8)	360 (16.6)	386 (17.8)
Very high	15 (0.3)	36 (1.1)	35 (1.9)	19 (1.1)	18 (0.4)	33 (0.8)	44 (2.4)	15 (0.8)	24 (0.5)	27 (0.7)	21 (1.0)	13 (0.6)	24 (0.5)	27 (0.7)	23 (1.0)	16 (0.7)
HDL																
Optimal	554 (11.2)	639 (19.2)	336 (19.9)	352 (19.2)	424 (9.9)	769 (19.2)	361 (19.5)	356 (19.2)	576 (12.9)	617 (16.2)	319 (15.6)	331 (16.2)	582 (12.8)	611 (16.5)	340 (15.7)	357 (16.5)
Border	1605 (32.5)	1322 (39.7)	712 (38.9)	728 (39.7)	1335 (31.3)	1592 (39.8)	706 (38.1)	737 (39.8)	1528 (34.3)	1399 (36.7)	738 (36.0)	752 (36.7)	1581 (34.7)	1346 (36.3)	820 (37.8)	786 (36.3)
High risk	2780 (56.3)	1367 (41.1)	754 (41.2)	752 (41.1)	2507 (58.8)	1640 (41.0)	785 (42.4)	759 (41.0)	2354 (52.8)	1793 (47.1)	990 (48.4)	964 (47.1)	2392 (52.2)	1755 (47.3)	1008 (46.5)	1025 (47.3)
LDL																
Optimal	2823 (57.2)	1380 (41.5)	779 (42.5)	760 (41.5)	2337 (54.8)	1866 (46.6)	843 (45.5)	864 (46.6)	2387 (53.5)	1816 (47.7)	969 (47.3)	976 (47.7)	2515 (55.2)	1688 (45.5)	969 (44.7)	986 (45.5)
Border	1625 (32.9)	1344 (40.4)	730 (39.9)	740 (40.4)	1425 (33.4)	1544 (38.6)	693 (37.4)	714 (38.6)	1547 (34.7)	1422 (37.3)	767 (37.5)	764 (37.3)	1542 (33.9)	1427 (38.4)	843 (38.9)	833 (38.4)
High risk	491 (9.9)	604 (18.1)	323 (17.6)	332 (18.1)	504 (11.8)	591 (14.8)	316 (17.1)	274 (14.8)	524 (11.8)	571 (15.0)	311 (15.2)	307 (15.0)	498 (10.9)	597 (16.1)	356 (16.4)	349 (16.1)

GGT: gamma-glutamyl transferase; ALT: alanine aminotransferase; AST: aspartate aminotransferase; ALP: alkaline phosphatase; Chol: cholesterol; TG: triglyceride; HDL: high-density lipoprotein; LDL: low density lipoprotein

**Expose: People with high liver enzymes ***Unexposed: People with Low liver enzymes

**Fig. 2:**
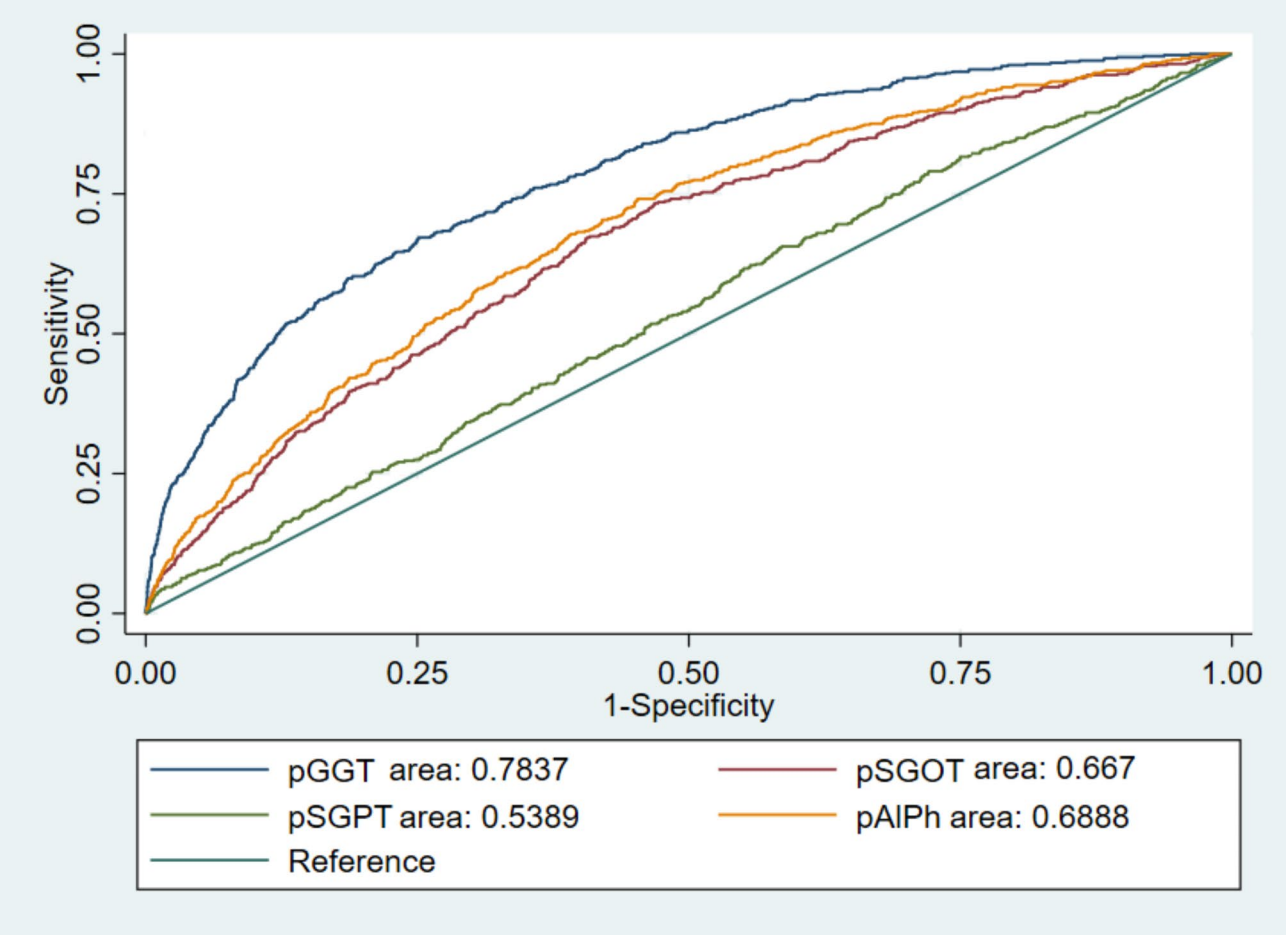
Receiver Operating Characteristic (ROC) for liver enzymes

Cut off point, accuracy, sensitivity, specificity, positive likelihood ratio, and negative likelihood ratio for liver enzymes are shown in Table 3. In ROC analysis, GGT with a cut-off value 21.8 U/L, a sensitivity of 50.0%, a specificity of 61.0%, LR + of 1.28, LR – of 0.81 an AUROC of 0.7837 had it has the highest quality of diagnostic value (accuracy) among liver enzymes Fig. 2.

**Table 3:** Cut off point, accuracy, sensitivity, specificity, positive likelihood ratio, and negative likelihood ratio for liver enzymes

Liver enzymes	Cut off	Aucracy	Sensetivity	Specificity	LR+	LR-
**GGT**	21.8	0.78	0.50	0.61	1.28	0.81
**ALT**	21.3	0.53	0.51	0.52	1.02	0.98
**AST**	20.0	0.66	0.52	0.53	1.02	0.98
**ALP**	194	0.68	0.50	0.55	1.12	0.91

GGT: gamma-glutamyl transferase; ALT: alanine aminotransferase; AST: aspartate aminotransferase; ALP: alkaline phosphatase

Also multivariate CPHM showed there is a relationship between participants with high level of GGT and had a higher risk of HTN (hazard ratio (HR) 1.34; 95% CI: 1.11–1.63) compared to those with low level of GGT. After PSM analysis, the effect of GGT on HTN remained positive and significant. The results showed probably that participants with high level of GGT had significantly higher risk of HTN (HR 1.48; 95% CI: 1.22–1.78) Table 4.

After PSM analysis, Pseudo-R2 decreased for all liver enzymes especially GGT, therefore, PSM produced better quality models for predicting HTN. GGT had greater predictive power relative to other liver enzymes in predicting HTN as measured by the AIC and BIC Table 4.

**Table 4:** Adjusted Hazard ratio (aHR) and 95% CI of the HTN according to the liver enzymes using CPHM and PSM analysis

Variables		Full population	Propensity score matching
		aHR (95% CI)	aHR (95% CI)
GGT	N0	1	1
	Yes	1.34(1.11–1.63)	1.48(1.22–1.78)
	p-value	0.004	0.002
Goodness of fit index	R^2^ peso	0.138	0.001
	AIC	3515.909	1497.967
	BIC	3711.570	1533.68
ALT	N0	1	1
	Yes	1.12(0.92–1.35)	1.07(0.98–1.17)
	p-value	0.236	0.136
Goodness of fit index	R^2^ peso	0.145	0.004
	AIC	8781.022	6497.967
	BIC	9153.064	7533.68
AST	N0	1	1
	Yes	1.07(0.89–1.29)	1.03(0.95–1.12)
	p-value	0.860	0.474
Goodness of fit index	R^2^ peso	0.071	0.006
	AIC	8777.768	687.967
	BIC	9142.791	5433.68
ALP	N0	1	1
	Yes	0.98(0.82–1.17)	0.99 (0.91–1.08)
	p-value	0.860	0.898
Goodness of fit index	R^2^ peso	0.040	0.002
	AIC	8780.724	457.967
	BIC	9152.766	6544.68

Abbreviation: aHR: Adjusted Hazard ratio ;ALT: alanine aminotransferase; AST: aspartate aminotransferase; GGT: gamma-glutamyl transferase; ALP: alkaline phosphatase

*Adjusted for variables: Age group; Gender; Residence Type; Education Years; Socio-economic status; Physical Activity ; Alcohol Consumption; cholesterol; triglyceride; high-density lipoprotein; low-density lipoprotein; body mass index; Smoking status; Healthy Nutrition Index; Depression; diabetes mellitus; cardiovascular disease; Has Job; Salt Use; Oil Type; Family medical history.

The standardized mean difference for confounding variables between the two comparison groups (intervention) was higher than 10% after PSM. The balance in the distribution of confounding variables is equal between the two comparison groups Table 5.

**Table 5:** The standardized mean difference after propensity score matching

Variable name	The standardized mean difference
Age group	**0.28**
Gender	**0.26**
Residence type	**0.17**
Education Years	**0.16**
Socio-economic status	**0.13**
Physical activity	**0.11**
Alcohol consumption	**0.18**
Cholesterol	**0.14**
Triglyceride	**0.14**
High-density lipoprotein	**0.14**
Low-density lipoprotein	**0.15**
Body mass index	**0.15**
Smoking status	**0.19**
Healthy Eating Index	**0.25**
Depression	**0.25**
Diabetes mellitus	**0.13**
Cardiovascular disease	**0.25**
Job	**0.12**
Salt use	**0.11**
Used oil type	**0.34**
Family hypertension	**0.11**

**Fig. 3:**
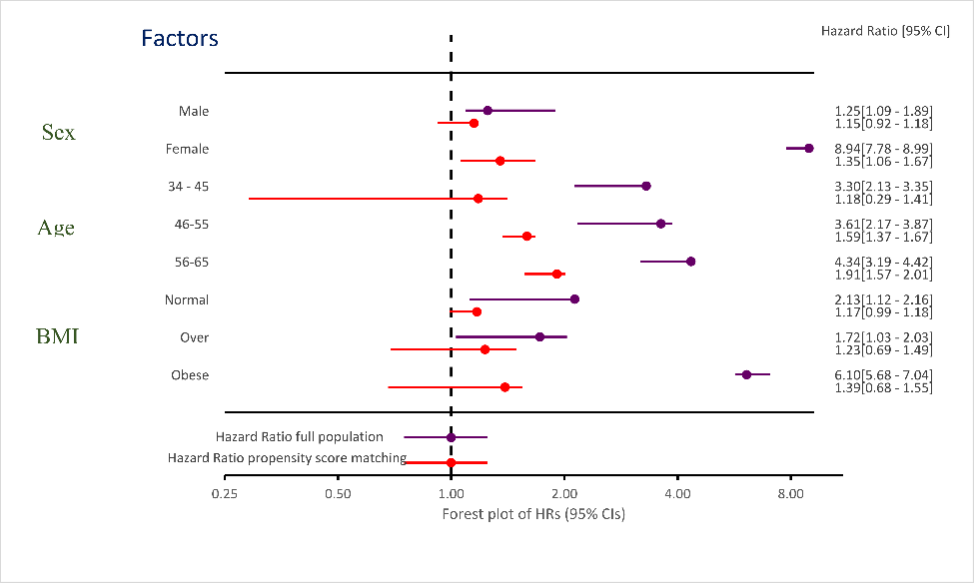
Forest plot of HR (95% CIs) before and after PSM for Age, BMI, and sex

After adjusting for 21 variables, gender, age, and body mass index (BMI) were significant (PV < 0.05). Figure 3 show forest plot of HR (95% CIs) before and after PSM analysis for age, BMI, and sex. After PSM analysis, HR for men and women were 1.15 and 1.35, HR for normal, overweight, and obese were 1.7, 1.23, and 1.39, and HR for 34–45, 46–55, and 56–65 age groups were 1.18, 1.59, and 1.91, respectively decreased. Therefore, by properly propensity score matching, the effect of important confounding variables on the outcome can be prevented.

## Discussion

In the present study, we investigated the associations between liver enzymes and the risk of HTN, in Kurdish adults. First of all, The results of multivariate CPHM showed There is a relationship between participants with high level of GGT and had a higher risk of HTN compared to those with low level of GGT. However, this association may be greatly influenced by various confounding factors such as age, gender, marital status, BMI, education and etc. Therefore, one-to-one PSM analysis performed to minimize the effects of confounding factors. After PSM analysis, impose of GGT on HTN risk significantly elevated. PMS effect was not observed for other enzymes. ALT and AST could increase the risk of HTN; however, these associations were not significant. Also, after PSM analysis, the effect of ALT and AST on HTN was decreased. Lastly, there was no relationship between ALP and HTN.

Our present findings of a positive association between HTN and GGT activities are confirmed previous results that showed the GGT activity had effect on elevated HTN risk significantly[21, 28]. Furthermore, the current results accordance with other studies reports. It has been showed that GGT level is positively associated with increases in Odd Ratio for HTN [29]. Rahman et al. [13] and Park et al. [30] showed that the serum GGT activity had an independent correlation with HTN and they are elevated in hypertensive persons. Two longitudinal studies showed that baseline serum GGT was an independent risk factor for HTN development [31]. Dan et al. have also reported a positive association of GGT with HTN in Indian adults [32]. Kotani et al. reported a positive association between higher serum GGT level and clinical HTN [33]. Cheung et al. showed that GGT was associated with incident HTN in Hong Kong Chinese [34].

The main mechanisms that connect GGT with HTN are not fully clear; although, there are some possible justifications. First of all, GGT is known as a marker of oxidative stress and inflammation [29]. Lee et al. conducted a longitudinal, multicenter epidemiologic study, they found that GGT is a predictor of incident hypertension and it is positively related to inflammation markers like fibrinogen, High-sensitivity C - reactive protein (CRP), and F2-isoprostane [35]. Secondly, GGT has been detected inside atherosclerotic plaques and it has been found that plays an important role in the pathogenesis of atherosclerosis, and also triggering low-density lipoproteins (LDLs) oxidation and other pro-oxidant reactions [36]. Third, GGT plays a central role in glutathione homeostasis [35, 37]. Therefore, increased GGT levels might be a marker of inflammation and oxidative stress, which are main features of HTN and CVDs.

Serum ALT, AST, and ALP did not indicate a significant association with HTN in the current investigation. Our results for ALT, AST, and ALP are in agreement with the findings of previous studies. Rahman et al. found that serum AST and ALP did not show a significant association with HTN, however, they reported ALT were significantly associated with HTN in Bangladeshi adults [13]. Gupta et al. found that there was no significant relationship between liver enzymes (ALT, AST and GGT) and HTN [38]. In contrast, some studies found that ALP is associated with HTN [39, 40]. Various reference values, age range, demographics characteristics, ethnicity, and etc. might be significant factors for the observed variations of these studies.

### Limitation and strength

There are some limitations in this study. Assessment of ALP, ALT, AST, and GGT in plasma can be non-specific and found in other diseases hepatitis B and C, biliary diseases, musculoskeletal diseases, and myocardial injury. The diagnostic performance of GGT in this study is good but there is no external data for extrapolation and verification of results. Despite these limitations, this study is the first investigation, to the best of our knowledge, to assess the association between liver enzymes and HTN using PSM analysis via a follow-up cohort study in an Iranian population in the Kurdish region. Major strengths of this study are the large prospective design, the length of follow-up, and the standardized protocol. Another strength of the present study included applying PSM analysis to eliminate the effects of confounding factors including age, BMI, lipids, smoking, physical activities and etc. to examine the relationship between HTN and liver enzymes.

## Conclusion

In conclusion, the higher serum GGT level was positively associated with the prevalence of HTN in Kurdish adults. This study showed that GGT has a potential diagnostic value for HTN. Therefore, monitoring GGT levels is helpful in the early detection of HTN. Further studies are needed to confirm the mechanisms between increased liver enzymes and develop HTN in the general population.

## Electronic supplementary material

Below is the link to the electronic supplementary material.

Supplementary Material 1: Association between Serum Liver Enzymes and hypertension using Propensity Score Matching Analysis: Evidence from a Large Kurdish Cohort

## Data Availability

The datasets used and/or analyzed during the current study are available from the corresponding author on reasonable request. All the information on how to access the RaNCD public data archive, with a list of current proposals and papers under preparation, can be found on our website: www.persiancohort.com.
