# Supplementary material for: Association between serum liver enzymes and hypertension using propensity score matching analysis: evidence from a large kurdish prospective cohort study
Source: BMC Cardiovasc Disord. 2022 Nov 10;22:476. doi: 10.1186/s12872-022-02884-3 (PMC9647908; doi:10.1186/s12872-022-02884-3)
Supplement: Supplementary file 1 — Supplementary Material 1: Association between Serum Liver Enzymes and hypertension using Propensity Score Matching Analysis: Evidence from a Large Kurdish Cohort [file 12872_2022_2884_MOESM1_ESM.docx]

**Supplementary information for**

**Association between Serum Liver Enzymes and hypertension using Propensity Score Matching Analysis: Evidence from a Large Kurdish Cohort**

**S1** Basic demographic data of the study population [n (%)] base on liver enzymes and variables (Residence Type; Socio-economic status; Healthy Nutrition Index; Depression; Has Job; Used Oil Type)

| Variables | GGT | | | | ALT | | | | | AST | | | | | | ALP | | | |
| --- | --- | --- | --- | --- | --- | --- | --- | --- | --- | --- | --- | --- | --- | --- | --- | --- | --- | --- | --- |
|  | Ful  l population | | Propensity score matched | | Full  population | | propensity score matched | | | Full population | | | propensity score matched | | | Full population | | propensity score matched | |
|  | Un*** expose | Expose** | Un expose | Expose | Un  expose | Expose | Un  expose | Expose | | Un  expose | | Expose | Un  expose | | Expose | Un expose | expose | Un expose | expose |
|  | 4939 | 3328 | 1832 | 1832 | 4266 | 4001 | 1852 | 1852 | | 4458 | | 3809 | 2047 | | 2047 | 4555 | 3712 | 2168 | 2168 |
| Residence Type | | | | | | | | | | | | | | | | | | | |
| City | 2774  (56.2) | 2162  (65.0) | 1176  (64.2) | 1190  (65.0) | 2297  (53.8) | 2639  (66.0) | 1195 (46.5) | 1221 (65.9) | | 2788  (62.5) | | 2148  (56.4) | 1138  (55.6) | | 1155  (56.4) | 2643  (58.0) | 2293  (61.8) | 1363  (62.9) | 1339 (61.8) |
| Rural | 2165  (43.8) | 1166  (35.0) | 656  (35.8) | 642  (35.0) | 1969  (46.2) | 1362  (34.0) | 657  (35.5) | 657  (35.5) | | 1670  (37.5) | | 1661  (43.6) | 909  (44.4) | | 892  (43.6) | 1912  (42.0) | 1419  (38.2) | 805  (37.1) | 829  (38.2) |
| SESq | | | | | | | | | | | | | | | | | | | |
| The poorest | 1085  (22.0) | 514  (15.4) | 287  (15.7) | 283  (15.4) | 1025  (24.0) | 574  (14.3) | 271  (14.6) | 266 (14.4) | | 835  (18.7) | | 764  (20.1) | 373 (18.2) | | 411  (20.1) | 859  (18.9) | 740  (19.9) | 419  (19.3) | 432  (19.9) |
| Poor | 1055  (21.4) | 555  (16.7) | 326  (17.8) | 305  (16.7) | 971  (22.8) | 639  (16.0) | 354  (19.2) | 296  (16.0) | | 926  (20.8) | | 684  (18.0) | 426 (20.8) | | 367  (17.9) | 886 (19.4) | 724  (19.5) | 429  (19.3) | 423  (19.5) |
| Medium | 992  (20.1) | 679  (20.4) | 351  (19.2) | 374  (20.4) | 860  (20.2) | 811  (20.3) | 348  (18.8) | 375  (20.2) | | 935  (21.0) | | 736  (19.3) | 405  (19.8) | | 396  (19.4) | 894 (19.6) | 777  (20.9) | 426  (19.7) | 454 (20.9) |
| Rich | 943  (19.1) | 728  (21.9) | 422  (23.0) | 401  (21.9) | 755  (17.7) | 916  (22.9) | 393  (21.2) | 424  (22.9) | | 916  (20.5) | | 755  (19.8) | 408 (19.9) | | 406  (19.8) | 919 (20.2) | 752  (20.3) | 437  (20.2) | 439  (20.3) |
| The richest | 864  (17.5) | 852  (25.6) | 446  (24.3) | 469  (25.6) | 655  (15.3) | 1061  (26.5) | 486  (26.2) | 491  (26.5) | | 846  (19.0) | | 870  (22.8) | 435  (21.3) | | 467  (22.8) | 997  (21.9) | 719  (19.4) | 458  (21.1) | 420  (19.4) |
| HEI | | | | | | | | | | | | | | | | | | | |
| Poor | 2562  (51.9) | 1604  (48.2) | 903  (49.3) | 883  (48.2) | 2294  (53.8) | 1872  (46.8) | 876  (47.3) | 867  (46.8) | | 2230  (50.0) | | 1936 (50.8) | 1063 (51.9) | | 1041  (50.8) | 2290  (50.3) | 1876  (50.6) | 1070  (49.9) | 1096  (50.5) |
| Need to be corrected | 1360  (27.5) | 1005  (30.2) | 521  (28.4) | 553  (30.2) | 1161  (27.2) | 1204 (30.1) | 565  (30.5) | 557  (30.1) | | 1277  (28.7) | | 1088  (28.6) | 580 (28.4) | | 585  (28.6) | 1287  (28.2) | 1078  (29.0) | 621  (28.6) | 630  (29.1) |
| optimal | 1017  (20.6) | 719  (21.6) | 408  (22.3) | 396  (21.6) | 811  (19.0) | 925  (23.1) | 411  (22.2) | 428 (23.1) | | 951  (21.3) | | 785 (20.6) | 404 (19.7) | | 422  (20.6) | 978 (21.5) | 758  (20.4) | 477  (22.0) | 443  (20.4) |
| Depression | | | | | | | | | | | | | | | | | | | |
| No | 4826  (97.7) | 3249  (97.6) | 1778  (97.6) | 1789  (97.6) | 4140  (97.1) | 3935 (98.3) | 1818  (98.2) | 1821  (98.3) | | 4331  (97.1) | | 3744  (98.3) | 2014  (98.4) | | 2012 (98.3) | 4456  (97.8) | 3619  (97.5) | 2113  (97.5) | 2114  (97.5) |
| yes | 113  (2.3) | 79  (2.4) | 44  (2.6) | 43  (2.4) | 126  (2.9) | 66 (1.7) | 338 (1.8) | 31  (1.7) | | 127  (2.1) | | 65 (1.7) | 33  (1.6) | | 35 (1.7) | 99 (2.2) | 93  (2.5) | 55  (2.5) | 54  (2.5) |
| Has Job | | | | | | | | | | | | | | | | | | | |
| No | 2671  (54.1) | 1792  (53.9) | 974  (53.2) | 987  (53.9) | 2364  (55.4) | 2098  (52.4) | 988  (53.3) | | 971  (52.4) | | 2348  (52.7) | 2115  (55.5) | | 1134  (55.4) | 1137  (55.5) | 2416  (53.0) | 2047  (55.1) | 1180  (54.4) | 1195  (55.1) |
| yes | 2268  (45.9) | 1536  (46.1) | 858  (46.8) | 845  (46.1) | 1901  (44.6) | 1903  (47.6) | 864  (46.7) | | 881  (47.6) | | 2110  (47.3) | 1694  (44.5) | | 913  (44.6) | 913  (44.6) | 2139  (47.0) | 1665  (44.9) | 988  (45.6) | 973  (44.9) |
| Used Oil Type | | | | | | | | | | | | | | | | | | | |
| Solid | 890  (18.0) | 615  (18.5) | 339  (18.5) | 339  (18.5) | 752  (17.6) | 753  (18.8) | 340  (18.4) | | 349 (18.8) | | 801  (18.0) | 704 (18.5) | | 354 (17.3) | 379 (18.5) | 822 (18.1) | 683 (18.4) | 394 (18.2) | 399  (18.4) |
| Semi-solid | 675  (13.7) | 419  (12.6) | 249  (13.6) | 231  (12.6) | 581  (13.6) | 513  (12.8) | 247 (13.3) | | 238  (12.9) | | 619 (13.9) | 475  (12.5) | | 282  (13.8) | 255  (12.5) | 611 (13.4) | 483  (13.0) | 298 (13.8) | 282  (13.0) |
| Liquid | 251  (5.1) | 160  (4.8) | 95  (5.2) | 88  (4.8) | 203  (4.8) | 208  (5.2) | 83 (4.5) | | 96  (5.2) | | 218  (4.9) | 193  (5.0) | | 95  (4.6) | 104  (5.0) | 222  (4.9) | 189  (5.1) | 107  (4.9) | 110 (5.1) |
| fried | 3103  (62.8) | 2120  (63.7) | 1141  (62.3) | 1167  (63.7) | 2714  (63.6) | 2509  (62.7) | 1176  (63.5) | | 1161  (62.7) | | 2805 (62.9) | 2418  (63.5) | | 1309  (64.0) | 1299  (63.5) | 2878 (63.2) | 2345  (63.2) | 1357  (62.6) | 1370  (63.2) |
| Other | 12  (0.2) | 12  (0.4) | 5  (0.3) | 7  (0.4) | 11  (0.3) | 13  (0.4) | 5  (0.3) | | 6  (0.13) | | 12  (0.3) | 12  (0.3) | 6  (0.3) | | 6  (0.3) | 17  (0.3) | 7  (0.2) | 9  (0.4) | 4  (0.2) |
| They do not fry food | 8  (0.2) | 2  (0.0) | 3  (0.1) | 1  (0.0) | 5  (0.1) | 5  (0.1) | 1  (0.0) | | 2  (0.1) | | 3  (0.0) | 7  (0.2) | 1  (0.0) | | 4  (0.2) | 5  (0.1) | 5  (0.1) | 3  (0.1) | 3  (0.1) |

GGT: gamma-glutamyl transferase; ALT: alanine aminotransferase; AST: aspartate aminotransferase; ALP: alkaline phosphatase; Residence Type; Socio-economic status; Healthy Nutrition Index; Depression; Has Job; Used Oil Type.

**Expose: People with high liver enzymes ***Unexposed: People with Low liver enzymes
